# Improved approach for the cryopreservation of mouse sperm by combining monothioglycerol and l-glutamine

**DOI:** 10.1016/j.cryobiol.2023.03.005

**Published:** 2023-03-29

**Authors:** Nguyen T. Van, Sangwon V. Kim

**Affiliations:** a Department of Microbiology and Immunology, Sidney Kimmel Medical College, Thomas Jefferson University, Philadelphia, PA, 19107, USA; b Sidney Kimmel Cancer Center, Jefferson Health, Philadelphia, PA, 19107, USA

**Keywords:** Monothioglycerol, MTG, l-glutamine, CARD method, Sperm, Cryopreservation, In vitro fertilization, IVF

## Abstract

The CryoPreservation Media (CPM) for mouse sperm using raffinose and skim milk have been improved by adding either monothioglycerol (MTG) or l-glutamine to reduce the oxidative damage during sperm freezing and thawing. The CARD-CPM utilizing l-glutamine, but not MTG, has been widely used to meet the rising demand for cryopreservation of genetically modified mice, as the CARD method also improved sperm capacitation and in vitro fertilization (IVF). However, the viability of sperm frozen in the CARD-CPM is highly variable, indicating a room for improvement. To develop a more dependable technique for mouse sperm cryopreservation, we investigate whether combining MTG and l-glutamine in the CPM (MG-CPM) can produce a synergistic impact on sperm thawing and IVF rate. We found that MG-CPM reduced the incidence of infertility and increased the IVF success rate. Therefore, cryopreservation of mouse sperm in MG-CPM is a reliable method to ensure embryo generation from frozen sperm.

## 1. Brief communication

Recent advances in the CRISPR/CAS-mediated modification of mouse genome have made it much easier to generate new lines of knock-out or knock-in mice. Consequently, more and more genetically modified mice have been created to facilitate biomedical research. However, housing mice is expensive, and it is ethically preferable to keep only the minimum number of mice required for active research. These facts raise the issue of preserving valuable resources, such as genetically modified mice, for future research.

**Fig. 1.**
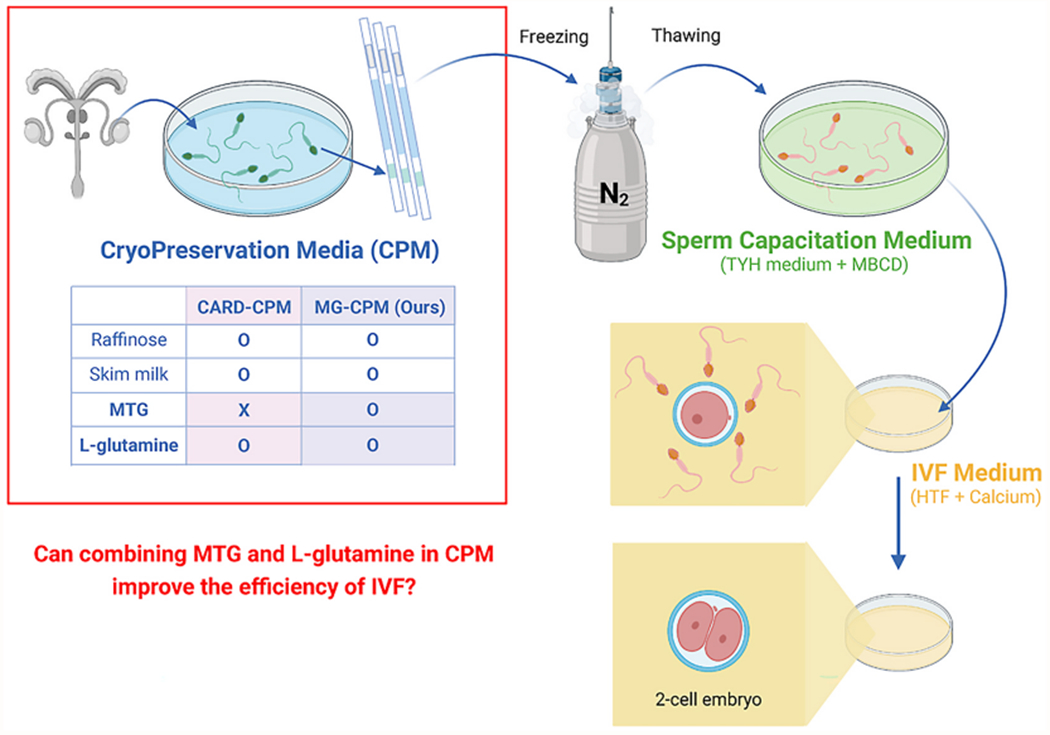
The main question and experimental design. The three media are used for the cryopreservation of mouse sperm and the subsequent production of embryos with thawed sperm using CARD method: Cryopreservation medium for sperm (CPM), sperm capacitation medium, and in vitro fertilization (IVF) medium. Here, we examined whether altering the composition of CPM improves the efficiency of embryo generation by thawed sperm (CARD-CPM vs. MG-CPM). The main question was presented in red text and a red box. The composition of the sperm capacitation medium and IVF medium remained unchanged from the CARD method. Figure was created by BioRender.com. MTG: monothioglycerol; TYH medium: Toyoda, Yokoyama, Hoshi medium; MBCD: methyl-β-cyclodextrin; HTF: human tubal fluid.

The cryopreservation of genetically modified mice can be accomplished by the cryopreservation of sperm, embryos, or eggs. Among these, sperm cryopreservation is the simplest method and can facilitate distribution, due to the small number of mice required and speedy procedures for obtaining live sperm. However, cryopreservation of mouse sperm has been more challenging than that of other species [5]. Fortunately, the discovery of raffinose as a cryoprotectant for mouse sperm in the 1990s made it possible to cryopreserve mouse sperm [5]. This method utilizing 18% raffinose and 3% skim milk as sperm cryopreservation media (CPM) was initially reported to result in inconsistency and low fertility of thawed sperm from mice with C57BL/6 background, which is used as a main strain for genetic modification [5]; this issue was only partially resolved by adding either monothioglycerol (MTG) or l-glutamine to reduce oxidative damages and the stress from thawing [3,7]. To further ensure the success of sperm cryopreservation, the conditions must be further optimized for in vitro fertilization (IVF) of mouse eggs with thawed sperm, as the technique of sperm cryopreservation is ineffective unless they can produce live mice later. There have been significant improvements in IVF techniques. First, sperm capacitation was facilitated by removing cholesterol from the sperm plasma membrane via treatment with methyl-β-cyclodextrin (MBCD) during sperm capacitation step after sperm thawing, as would normally occur in the oviduct in vivo [6]. Second, reducing glutathione in the IVF media prevented oxidative stress on the sperm and facilitated sperm penetration of eggs [8]. Combining the above-mentioned two improvements by MBCD and reducing glutathione with CPM containing 18% raffinose, 3% skim milk, and l-glutamine (CARD-CPM) [7], the CARD method of sperm cryopreservation and IVF (Fig. 1) was proposed and has become very popular among researchers [9,10].

**Fig. 2.**
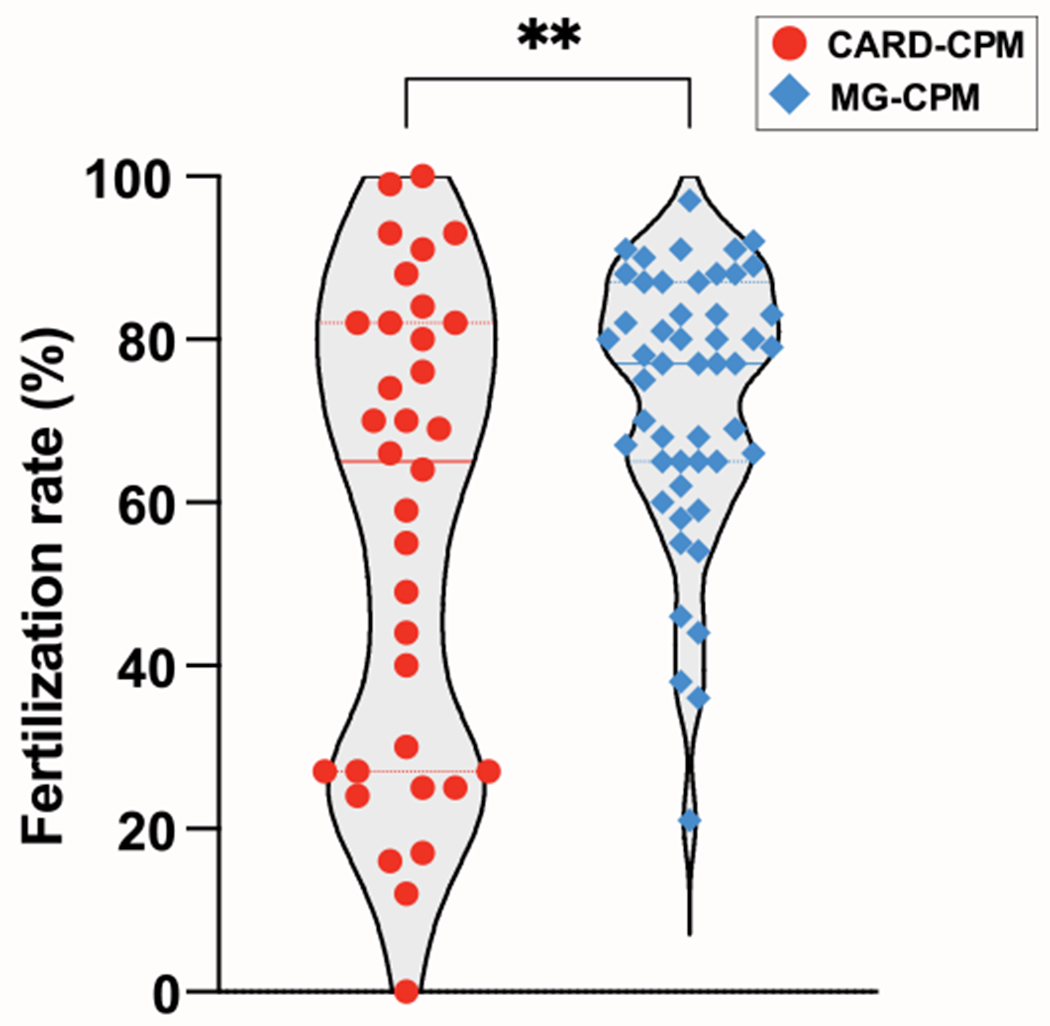
MG-CPM is a more reliable medium for sperm cryopreservation than CARD-CPM. Sperm that had been frozen in either CARD-CPM or MG-CPM was thawed and used for in vitro fertilization according to the CARD IVF protocol. Sperm and eggs from various mouse lines in a C57BL/6 background were used. At day 2, embryos reaching the 2-cell stage were counted from the total eggs utilized to calculate fertilization rate. Medians were labelled as solid line, and quartiles were labelled as dotted line. Average: 57.01% (CARD-CPM); 72.8% (MG-CPM). Sample size: n = 34 (CARD-CPM); n = 50 (MG-CPM); **: P < 0.0023 with student’s t-test.

Unfortunately, the CARD method of sperm cryopreservation occasionally has shown inconsistency in sperm survival or IVF success rate, which has been posited to be due to the differences in cooling rate at the time of cryopreservation [4]. We began working with mouse embryos and sperm in C57BL/6 background for cryopreservation in our laboratory and observed that, indeed, the CARD-CPM showed the occasional inconsistency in survival and a wide range of IVF success rates (Fig. 2). However, our effort to strictly regulate cooling rates during sperm freezing failed to eliminate this inconsistency in the IVF success rate. Therefore, we hypothesize that the inconsistency in CARD-CPM is likely due to the oxidative damage that sperm experience during thawing in the straws prior to being transferred to sperm preincubation media containing MBCD and IVF media containing reduced glutathione [1]. However, it has been shown that increasing the concentration of either one of antioxidants, MTG or l-glutamine in the CPM did not increase the survival of thawed sperm [2,3]. Therefore, we sought to determine whether a synergistic effect could be produced by combining MTG and l-glutamine in the CPM (MG-CPM) to improve the IVF success rate with thawed sperm under the CARD method of the sperm capacitation and IVF (Fig. 1).

The CARD-CPM composition is shown in Table 1 as previously described [10]. To make CARD-CPM (Table 1), 146 mg of l-Glutamine was placed in 10 mL of cell culture-grade water and vortexed for 3 min, followed by 3 min of incubation in 60 °C water bath. Subsequently, 1800 mg of D^+^ raffinose pentahydrate and 300 mg of dehydrated skim milk were added, and the solution was vortexed for 3 min. The solution was incubated further in 60 °C water bath for 90 min with 3 min of vortexing every 30 min. Next, the solution was centrifuged at 10,000 g for 60 min. The clear top portion of the supernatant was collected and filtered through a 0.22-μm filter. Final CARD-CPM solution was stored up to 3 months at room temperature. The quality of home-made CARD-CPM was confirmed by comparing it to commercially available CARD FERTIUP CPM (Cosmo Bio USA) during IVF. Our modified sperm cryopreservation media with both MTG and l-glutamine was named as MG-CPM (MTG + l-Glutamine CryoPreservation Medium). To make MG-CPM (Table 1), MTG (FW = 108.16; r = 1.295 g/mL) was diluted by placing 10 μL MTG to 2.5 mL of cell-culture–grade water, making a 44.7 mM solution of MTG. Then, 100 μL of 44.7 mM solution MTG was added to 10 mL of CARD-CPM and filtered through a 0.22-μm filter. Final solution of MG-CPM was stored at room temperature for up to 3 months.

For sperm cryopreservation, the following procedures were used for one sperm-donor mouse in C57BL/6 background using either CARD-CPM or our MG-CPM. First, 60 μL of cryoprotective reagents was placed into a 35-mm dish, covered with mineral oil, and another 60 μL of cryoprotective reagents was added to the same drop to increase the volume without spreading. The dish was incubated in a 37 °C incubator (5% CO_2_, 5% O_2_ balanced with N_2_) at least 30 min before sperm collection. A Styrofoam box (15”w x 14”d x 10”h) was filled with 6–9 cm height of LN_2_, and a small piece of Styrofoam (9” w x 7” d) was placed inside floating. A box lid was placed on top, allowing the box to be filled with vapor LN_2_ at least 30 min before sperm collection. Six 0.25 cc straws (Reproduction Provisions LLC) were prepared for each donor mouse. One sperm donor was euthanized by cervical dislocation; cauda epididymites and vas deferens were removed and immediately placed in the CPM dish taken out from the incubator. Sperm was released by making 6–7 cuts through the epididymis and vas deferens. The plate was rotated for 1 min to disperse sperm. The straw was connected to a syringe, and the contents were aspirated in the following order: 100 μL of HTF (Human Tubal Fluid), 15 mm of air, 10 μL of sperm suspension, 15 mm of air. The straw was sealed at both ends with heat sealer. Straws were put in the 145 mm flat cassettes (6 straws from one donor mouse in one cassette), and the cassettes were placed on top of Styrofoam float. The lid was closed, and the cassette was incubated in the vapor phase for 10 min. Then, the cassettes were dropped into the liquid nitrogen and transferred to LN_2_ tank for long-term storage.

Sperm capacitation medium (TYH [Toyoda, Yokoyama, Hoshi] medium with 0.75 mM MBCD) and IVF medium (HTF medium with 5.14 mM calcium) were prepared according to the CARD method [9]. For sperm capacitation medium, all reagents were added to cell-culture-grade water according to the order in the CARD method except two. PVA and CaCl_2_ were dissolved separately in water and added last. The final volume of the solution was adjusted and filtered through a 0.22-μm filter. Aliquots were stored at 4 °C. For IVF medium, all reagents were added and mixed to cell culture-grade water, except CaCl_2_ and BSA. CaCl_2_ was dissolved in water and added last. Next, the solution was gassed for 5 min with mixed gas (5% CO_2_, 5% O_2_ balanced with N_2_). BSA was added and allowed to dissolve without stirring. The final volume of the solution was adjusted and filtered through a 0.22-μm filter. Aliquots were stored at 4 °C for up to 3 months.

For sperm thawing and capacitation, the following steps were used: first, 90 μL of sperm capacitation medium was placed at the center of a 35-mm dish, covered with mineral oil. The dish was incubated in a 37 °C incubator for at least 30 min. A straw was removed from LN_2_ and immediately immersed into a 37 °C water bath. After 5 seconds, the straw was removed from the water bath and wiped with a tissue. Both sealed ends of the straw were cut off, and straws were connected to 1 ml syringe to push the thawed sperm onto 90 μL of sperm capacitation medium on the 35-mm dish. In vitro fertilization with thawed sperm was performed using IVF medium as described in the CARD method before [9,10].

After adding sperm to eggs for IVF dish, at 24 h we counted the number of embryos that reached 2-cell stage and calculated their percentage of the total eggs used. We demonstrated that sperm frozen in MG-CPM had a higher IVF success rate than those frozen in CARD-CPM (median 72% vs. 57%) (Fig. 2). Importantly, MG-CPM minimized extremely low fertilization rates, which must be eliminated for cryopreservation to be reliable (Fig. 2). We have not evaluated the efficiency of generating live pups from 2-cell embryos between CARD-CPM and MG-CPM due to our lack of capacity to perform sufficient embryo transfer procedures for data collection. However, it is highly likely that the efficiency of generating live pups from 2-cell embryos would be the same for both methods and we have confirmed that embryos produced from sperm frozen in MG-CPM are capable of producing live pups (data not shown). In addition, we occasionally observed under the microscope that some sperm thawed from CARD-CPM were extremely mobile in TYH-MBCD, whereas sperm thawed from MG-CPM were less mobile. However, these highly mobile sperm observed occasionally were only a very small portion of the thawed sperm from the same straw and did not have a significant impact on the overall fertilization rate under our IVF conditions with reduced glutathione (Fig. 2). Rather, we believe it is more important to minimize the risk of sperm damage during thawing to improve IVF efficiency, specifically to eliminate the possibility of very low fertilization rate (Fig. 2). Therefore, we propose here that for ensuring the success of IVF following sperm thawing, MG-CPM containing 18% raffinose, 3% skim milk, 447 μM MTG, and 100 mM l-glutamine is more reliable sperm cryopreservation medium than CARD-CPM containing 18% raffinose, 3% skim milk, 100 mM l-glutamine (Table 1).

## Figures and Tables

**Table 1:** Composition of Cryopreservation media (CPM).

Reagent	Source	CARD-CPM		MG-CPM	
		Quantity	Final Concentration	Quantity	Final Concentration
D^+^ raffinose pentahydrate	Sigma-Aldrich R7630	1800 mg	18%	1800 mg	18%
Dehydrated skim milk	BD Diagnostics 232100	300 mg	3%	300 mg	3%
Monothioglycerol	Sigma-Aldrich M6145			100 μL, of 44.7 mM	447 μM
l-Glutamine	Sigma-Aldrich G8540	146 mg	100 mM	146 mg	100 mM
Cell culture–grade water	Sigma-Aldrich W1503	10 mL		10 mL	
